# Biomechanical Comparison of Tibial Plateau Leveling Osteotomy Performed With a Novel Titanium Alloy Locking Plate Construct vs. an Established Stainless-Steel Locking Plate Construct

**DOI:** 10.3389/fvets.2021.698159

**Published:** 2021-09-08

**Authors:** Seth Bleakley, Ross Palmer, Nate Miller, Kirk McGilvray, Slobodan Tepic

**Affiliations:** ^1^CARE Surgery Center, Glendale, AZ, United States; ^2^Department of Clinical Sciences, Colorado State University, Fort Collins, CO, United States; ^3^Colorado Canine Orthopedics and Rehab, Colorado Springs, CO, United States; ^4^Kyon Veterinary Surgical Products, Kyon AG, Zurich, Switzerland

**Keywords:** TPLO, stifle, orthopedics, cranial cruciate ligament, locking plate, fixation

## Abstract

A novel canine tibial plateau leveling osteotomy (TPLO) fixation device was recently developed with design features such as titanium alloy (TA) material, distal monocortical screw fixation, and a point contact undersurface specifically targeted to reduce surgical site infection rates by ensuring tissue perfusion under the plate. The strength of the novel TPLO construct was compared with that of a predicate stainless steel (SS) locking plate construct with bicortical screws in 16 paired cadaveric canine limbs. The mean loads to failure were 716.71 ± 109.50 N (range 455.69–839.69 N) and 629.50 ± 176.83 N (range 272.58–856.18 N) in the TA and SS groups, respectively. The average ratio of the loads to failure of the paired specimens was 1.18 (*p* = 0.031). No failure of the TA constructs involved the distal fixation with monocortical screws. Substantial mechanical equivalence of this novel TA monocortical/bicortical fixation construct to an established SS bicortical screw fixation construct is demonstrated. Clinical investigation of potential merits of this novel TA, monocortical/bicortical locking screw/plate system is now warranted.

## Introduction

Rupture of the cranial cruciate ligament (CCL) is a common cause of hindlimb lameness and stifle osteoarthritis (OA) in dogs. In 2005, the annual cost of medical and surgical management of CCL rupture in the USA was estimated to be >$1.3 billion and has likely increased since (1, 2). Tibial plateau leveling osteotomy (TPLO) is one of the most commonly performed procedures for the stabilization of cranial cruciate ligament deficient stifles in dogs (3).

Surgical site infection (SSI) is one of the most common complications after TPLO (4–18). SSI undoubtedly has a negative impact on patient well-being, client satisfaction, and financial burden and often necessitates an additional anesthetic episode and surgical procedure for implant removal (19). Few of the TPLO fixation devices to date have sought to reduce surgical site infection (SSI) rates (4). A novel TPLO fixation device was recently developed with design features such as titanium alloy (TA) material, distal monocortical screw fixation, and a point contact undersurface specifically targeted to reduce SSI rates by ensuring tissue perfusion under the plate (20–33).

In the human orthopedic industry, it is commonplace for novel devices to be evaluated by the Federal Drug Administration (FDA) for “substantial equivalence” to a previously cleared device (a so-called predicate device) in lieu of testing in independent clinical trials. This FDA clearance mechanism is referred to as the 510K premarket notification process (34). With this established process in mind, we sought to evaluate a novel, titanium alloy (TA) distal monocortical locking screw/plate device for substantial mechanical equivalence to a predicate stainless steel (SS) bicortical locking/conventional screw/plate device before pursuing clinical studies to investigate its abilities to affect SSI rates. Our hypothesis for this phase I study was that there would be no difference in load to failure between the two fixation devices when evaluated as load-sharing TPLO implant–bone constructs applied to paired canine cadaveric hind limbs.

## Materials and Methods

Cadaveric paired pelvic limbs were harvested by disarticulation of the coxofemoral joint in eight mixed breed dogs (~20–30 kg) that were euthanatized for reasons unrelated to this study. The dogs had been frozen immediately (−20°C) after euthanasia and thawed at room temperature for 48 h before limb harvest. Limbs were then stored in a refrigerated environment (2–8°C) and tested within 48 h of thawing. All limbs were free of radiographically detectable disease. Limbs were randomly assigned *via* computer randomization to either a group with the novel TPLO plate (Kyon Veterinary Surgical Products, Zurich, Switzerland) construct (TA group) or a group with the predicate stainless steel plate (DePuy Synthes, New Brunswick, NJ, USA) construct (SS group). The contralateral limb of each cadaver was assigned to the opposite treatment group to minimize variability.

**Figure 1:**
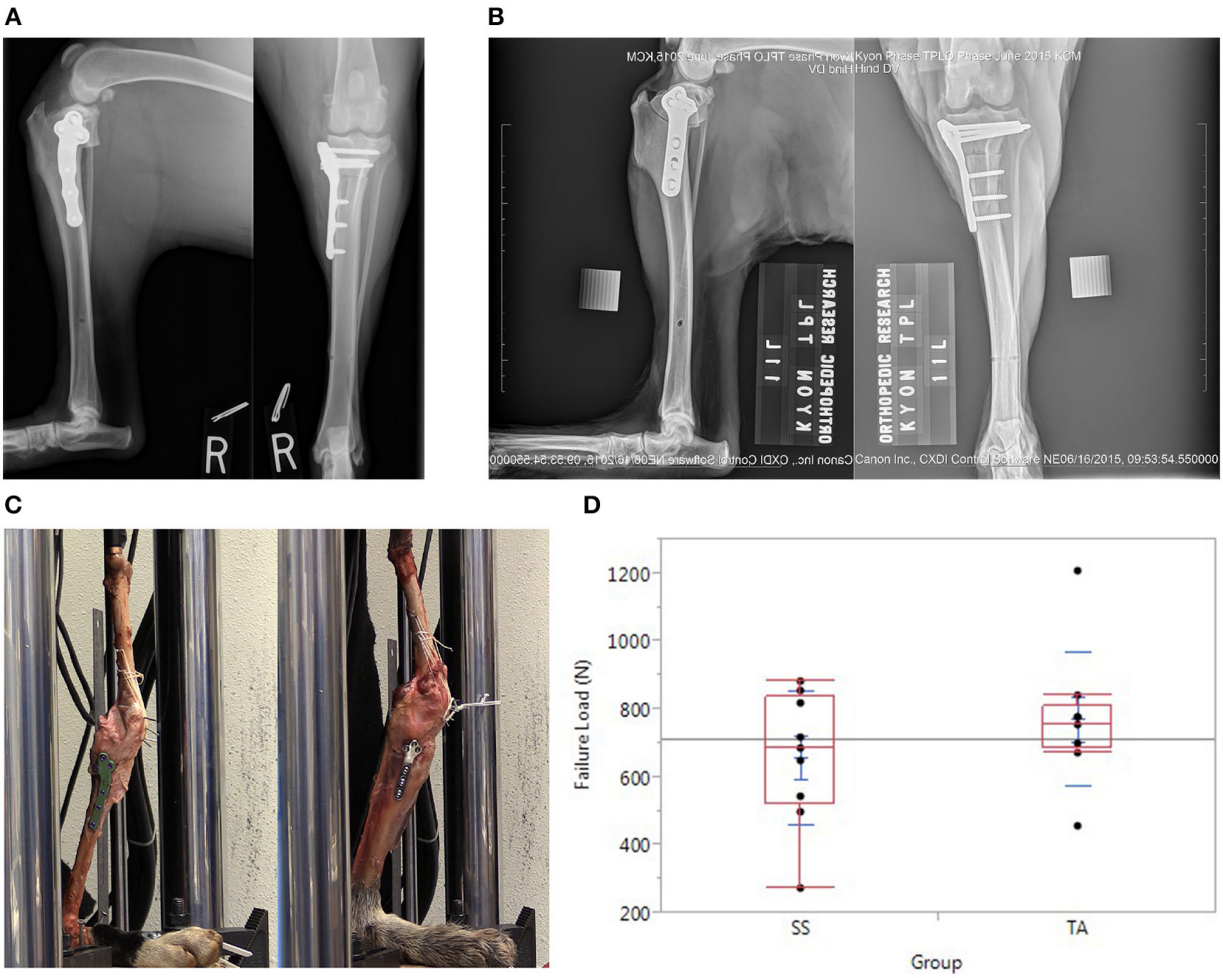
**(A)** Orthogonal postoperative radiographs of a stifle in the TA group demonstrating plate placement with monocortical screws in the distal segment in accordance with manufacturer guidelines. **(B)** Orthogonal postoperative radiographs of a stifle in the SS group. **(C)** TPLO constructs positioned in servo-hydraulic testing machine before testing. **(C)** Left is an example of a construct in the TA group. **(C)** Right is an example of a construct in the SS group. **(D)** Graph depicting the load at the time of failure after TPLO with an established stainless steel plate (SS group) vs. a novel titanium alloy plate with mono-cortical locking screws (TA group).

In the TA group, TPLO was performed by a board-certified surgeon (N.M.) experienced with the technique. Preoperative radiographic planning was performed to determine the desired rotation and saw blade positioning as for clinical cases. Care was taken to ensure adequate tibial tuberosity size cranial to the osteotomy (a minimum of 10 mm or as judged by the surgeon based off of patient size). A jig (Slocum Enterprises, Eugene, OR, USA) was used during the procedure. The distal jig hole was placed in the center of the tibial diaphysis to avoid a stress riser during testing. All screws were 4.0-mm-diameter titanium alloy locking screws; three of which in the distal segment were monocortical per manufacturer recommendations and three of which in the proximal segment were bicortical (Figure 1A). The osteotomy was compressed with a conventional 3.0-mm-diameter bicortical screw in the proximal hole after placement of the distal screws. After filling all screws, the 3.0-mm proximal conventional compression screw was replaced with a 4.0-mm bicortical locking screw per manufacturer guidelines.

In the SS group, routine TPLO was performed with an established stainless-steel locking TPLO plate by a board-certified surgeon (R.P.) experienced with the technique. A jig (Slocum Enterprises) was used during the procedure. The osteotomy and rotation were performed to match the contralateral limb. Three 3.5-mm-diameter bicortical conventional screws were placed in the distal fragment and three 3.5-mm-diameter bicortical locking screws were placed in the proximal segment using a manufacturer-recommended compression technique (Figure 1B). Bicortical screw fixation was assured, but screw length was not optimized (excessive screw length was accepted) to minimize screw inventory required for the study.

After removal of musculature, all limbs were tested in a servo-hydraulic testing machine with a 5,000-N force transducer (Mini Bionix 858 MTS Systems Corporation, Eden Prairie, MN, USA) to measure load to failure (Figure 1C). The limbs were positioned with the stifles at 135° to correspond with standing angle during mid-stance phase, the metatarsals were fixed with screws to a wooden base, and the load was applied to the femoral head along a line connecting the femoral head to the tarsus. The patella was fixed to the distal femur with multiple Kirschner wires drilled at an oblique angle and tied to a pair of K-wires inserted through the mid-shaft of the femur in the frontal plane by a high-strength braided ultra-high molecular weight polyethylene suture (PowerFiber; CP Medical Inc., Portland, OR, USA) to simulate “worst-case scenario” maximal quadriceps muscle group stiffness. This methodology also minimized inter-construct variability. All limbs were preconditioned for 10 cycles, 0.25 Hz with 5 mm of displacement before being ramped to failure at a rate of 1 mm/s until either implant failure or stifle collapse.

Statistical analyses were performed at the Clinical Trial Unit of the University Hospital, Basel, Switzerland. Software used included *R* version 3.6.3, xtable_1.8-3, plyr_1.84, reshape_0.85, and lattice_0.20-38. Because the small sample size (eight limb pairs) did not permit assessment of distribution (used for a parametric test) or the symmetry around 1 of the data, a randomization test was performed. The geometric mean and a 95% bootstrap normal CI were calculated for the ratios in the observed data. The CI was estimated using 999 bootstrap repetitions. Normality of the bootstrap repetitions was assessed visually using a Normal Q–Q plot.

## Results

The mean loads to failure were 716.71 ± 109.50 N (range 455.69–839.69 N) and 629.50 ± 176.83 N (range 272.58–856.18 N) in the TA and SS groups, respectively (Figure 1D). Modes of failure are recorded in Table 1 and included tibial tuberosity fracture (5), patellar tendon avulsion (4), lateral construct collapse (2), medial collateral ligament rupture (2), fibular fracture (1), tibial fracture (1), and screw pull-out (1). The case with screw pull-out was in the SS group and had bicortical screws which pulled out of the proximal segment in addition to a fracture at the tibial tuberosity. In Table 2, the results of the randomization test and bootstrap normal CI are presented. The average ratio of the loads to failure of the paired specimens was 1.18 (*p* = 0.031). The 95% CI of the ratio between the strength of limbs operated with the two plates was [0.99; 1.27].

**Table 1:** Load at failure and modes of failure in paired cadaveric TPLO constructs in the titanium alloy (TA) and stainless steel (SS) groups.

**Dog—leg**	**Breed**	**Group**	**Load at failure (N)**	**Mode of failure**
1—right	Pitbull	TA	756	Patellar tendon avulsion at the patella
1—left	Pitbull	SS	543	Medial collateral ligament rupture
2—right	Pitbull	TA	674	Lateral construct collapse
2—left	Pitbull	SS	649	Lateral construct collapse
3—right	Pitbull	TA	840	Patellar tendon rupture
3—left	Pitbull	SS	817	Patellar tendon avulsion at patella
4—right	Pitbull	SS	497	Tibial tuberosity fracture
4—left	Pitbull	TA	698	Lateral collateral ligament rupture and tibial tuberosity avulsion
5—right	Labrador mix	SS	718	Patellar tendon avulsion at patella
5—left	Labrador mix	TA	779	Patellar tendon avulsion at patella
6—right	Pitbull	SS	684	Tibial tuberosity avulsion
6—left	Pitbull	TA	776	Tibial tuberosity avulsion
7—right	Pitbull	SS	273	Medial collateral ligament rupture
7—left	Pitbull	TA	456	Lateral collapse
8—right	German Shepherd Dog	TA	756	Fibular fracture and lateral collapse
8—left	German Shepherd Dog	SS	856	Tibial tuberosity fracture and 3 proximal screws pulled out with cranial fracture

**Table 2:** Results of the randomization test and bootstrap normal CI.

Extreme observations	8
Total number of combinations	256
*P-*value (randomization test)	0.031
Geometric mean of observed ratios	1.18
Bootstrap CI	[0.99; 1.27]

## Discussion

Our results rejected the hypothesis that there would be no difference in load to failure between the two fixation devices. The ratio of load to failure was significantly greater in the TA group compared with the SS group (*p* = 0.031) under the unique loading-sharing cadaveric conditions of this experiment. No failure of the TA constructs involved the distal fixation with monocortical screws. Substantial mechanical equivalence of the novel TA construct with the predicate SS construct was confirmed in this single load to failure testing model.

The advent of locking plates has permitted stable fixation with monocortical screws. Conventional bone plates rely on friction between the plate and the bone that is generated by compression of the screw head on the bone plate. Failure of conventional plates is often attributed to screw loosening and axial pull-out (35). Failure of locking systems requires concurrent axial pull-out of all screws or compressive failure of the bone surrounding the screws (35). The force or load required to cause failure of all screws greatly exceeds that required to cause failure in sequential fashion (35). Therefore, a locking construct requires engagement of fewer cortices than a non-locking to achieve similar load resistance. In a study of 30 Greyhound femurs, Field et al. (36) demonstrated that axially loaded locking monocortical plate-rod constructs conferred no difference biomechanically to those employing locking bicortical screws. In our study, no construct failures were attributed to the monocortical screws. However, the monocortical screws were only placed in the diaphyseal bone of the tibia distal to the osteotomy per manufacturer guidelines. Bicortical screws are still recommended in the softer metaphyseal bone proximal to the osteotomy, although evidence to support this is currently lacking.

The effect of implant design on SSI after internal fracture fixation has been investigated. In 1993, the AO Institute developed a novel bone plate called the point contact fixator (PC-Fix) (24, 29, 30). The PC-Fix was the first internal fixator and employed point contact only on the bone surface as well as angle stability of screws achieved by a conical connection between screw heads and screw holes (24, 29, 30). The goal of this design was to reduce the impact of implant placement on periosteal blood supply. In addition, the locking design permitted stability with monocortical screw placement to theoretically reduce disruption to endosteal blood supply. The result was faster healing and reduced infection rates when compared with the dynamic compression plate. In a study of rabbit tibiae inoculated with *Staphylococcus aureus* and fixated with either the PC-Fix plate or a dynamic compression plate (DCP), Arens et al. (27) demonstrated a lower infection rate (26%) with the PC-Fix when compared with the DCP (63%).

The Advanced Locking Plate System (ALPS; Kyon Veterinary Surgical Products, Zurich, Switzerland) is a locking plate system based on the PC-Fix design developed exclusively for veterinary use. ALPS plates are made from c.p. titanium which has been shown to reduce infection rate when compared with stainless steel in a rabbit study (25). As both the PC-Fix and DCP were manufactured from c.p. titanium, the reason for a lower infection rate was attributed to implant design and independent of implant material. In a prospective multi-center study of 1,229 PC-Fixators placed in human patients, Eijer et al. (28) reported an infection rate of 1.1%, lower than that reported with DCP. The difference in implant design is multi-factorial and could include a reduced disruption of periosteal blood supply secondary to the point contact design, a reduced disruption endosteal blood supply secondary to monocortical screw fixation, and/or the use of locking screws. Which factor has the biggest effect on infection rate is not clear and requires further investigation. Maintenance of blood supply theoretically reduces infection by delivering inflammatory cells and mediators, key to an effective immune response. Whether endosteal blood supply or periosteal blood supply is more important in the face of infection is unknown, nor has the effect of monocortical vs. bicortical screw placement on blood supply to cortical bone been investigated.

Locking screws have been shown to reduce infection rates in TPLO constructs (4). In a retrospective study of 208 dogs that underwent TPLO using a variety of bone plates, non-locking constructs were associated with a higher incidence of infection (*p* = 0.01) (4). The authors hypothesized that the protective effect of the locking plate is due to lack of disruption of the underlying cortical bone perfusion in comparison with conventional plates (which compress the undersurface of the plate to the cortical bone), the decreased incidence of inflammatory complications from loosening of the hardware (loose hardware has been shown to propagate an inflammatory response and promote infection) (37) and a faster rate of bone healing because of more stable fixation (with stimulation of primary rather than secondary bone healing) (10, 12). Seeing that implant design can influence the incidence of postoperative TPLO SSI, investigation to identify other implant features that may further reduce SSI seems warranted.

The novel bone plate investigated in our study is manufactured from a titanium aluminum vanadium alloy (TAV). The screws are manufactured from a titanium aluminum niobium alloy (TAN). Both of these alloys have higher static and fatigue strengths than commercially pure (c.p.) titanium or stainless steel (38). Titanium alloys also have a lower modulus of elasticity compared with stainless steel, and thus much higher strain tolerance, both under static and cyclic loading (38).

Biological benefits of titanium alloy include a lower infection rate when compared with stainless steel (23, 25, 32). A possible reason for this is the fact that soft tissue adheres firmly to titanium-implant surfaces (21, 22), while a known reaction to steel implants is the formation of a fibrous capsule, enclosing a liquid filled void (21, 22). Bacteria can spread and multiply freely in this non-vascularized space, which is also less accessible to the host defense mechanisms. Reports on the use of titanium alloy implants in dogs are limited. In a study of 282 fixations using ALPS, Nojiri et al. (33) reported an infection rate of 1.1%. Two of the three infection complications were from 11 cases known to have been infected at the time of surgery. Only one additional infection occurred in the remaining 271 cases. The infection rate associated with similar surgeries in dogs using other stainless steel systems is 5.2–21.3% (5–7).

Small sample size of our pilot study is a limitation which reduces statistical inference. While statistical significance was demonstrated, a small sample size increases the probability of a type 1 error and results should therefore be interpreted cautiously. TPLO was performed by a different board-certified surgeon in each group. This is limitation inasmuch as surgeon influence on results cannot be excluded.

There are a number of limitations related to the *ex vivo* nature of our study that should be considered when extrapolating our results to clinical applications. The constructs were not tested in a non-load-sharing model; therefore, the mechanical performance of the implants cannot be isolated from the mechanical contributions from the reconstructed bone. Kloc et al. (39) tested novel TPLO plates using an axially loaded gap model. A 3.2-mm osteotomy gap was maintained in all constructs in that study and load, axial displacement, and failure mode were recorded. This model permitted comparison of the isolated implants. While our model compares the strengths of TPLO constructs as they are often performed clinically, it permitted load sharing between the implants and the bone as a confounding factor. Variables in construct strength could result from surgical technique, surgeon, the level of compression achieved, and variation in bone strength. Conversely, a gap model fails to reflect a clinical construct. TPLO is typically performed with either a compressed osteotomy, or as in cases of alignment correction, some degree of load-sharing *via* some cortical contact at the osteotomy. The goal of our model was to more closely mimic a clinical situation by applying implants using a technique resembling clinical application. Efforts were made to minimize variables by applying all plates in compression mode and by matching surgical planning between contralateral limbs. Comparison of constructs using paired limbs reduced variables in bone strength. Our model permitted demonstration of mechanical adequacy in a clinically relevant construct.

There were no muscles on the tested constructs and the complex forces associated with walking were not replicated. Our method of modeling the quadriceps–patella–patellar ligament mechanism's ability to maintain stifle standing angle under load does not perfectly replicate the mechanism's dynamic response to progressive load application under physiologic conditions. There have been other intact stifle cadaveric models used in previous studies in canines (40, 41) and felines (42–45) that made efforts to account for the common calcanean tendon and quadriceps mechanism, as well as various standing angles (39). Evidence to our imperfect modeling is that the construct's mode of failure was generally different than what has been reported clinically (8–12). While it can be argued that cyclic loading applied to a more dynamically modeled quadriceps–patella–patellar ligament mechanism would have been more clinically relevant, perfect *ex vivo* modeling of the complex forces associated with muscle tension, weight bearing, and standing angle with various activities is not possible. We feel that the mechanical testing performed yielded relevant results; for if the novel plate/screw system had underperformed relative to the predicate in this format, implant design modifications may have been warranted before progressing to more costly and time-consuming cyclical testing protocols.

Only one observed failure could be attributed to the implant construct. This case involved screw pull-out and fracture associated with the proximal tibia in a dog in the SS group with bicortical locking screws in the proximal segment. Other modes of failure associated with the bone or soft tissues of the intact stifle could be independent of the construct group which is a limitation of our study. While observed modes of failure may reduce inference regarding isolated implant strength, the paired nature of the study design still permits comparison of overall construct integrity. The fact that there was no evidence of failure associated with the monocortical screws in the TA group was interesting.

There are limitations inherent with the cadaveric nature of our study. The lower temperature and lack of blood supply in cadaveric bone may affect fracture behavior and therefore not reflect *in vivo* bone. The limbs in our study were frozen and thawed before testing. It is possible that this could also have affected the mechanical properties of the bone. Mulargia et al. (46) investigated fracture and fatigue in osteocytes after cyclical loading of bovine bone specimens and showed freezing bone samples did not affect the fatigue behavior or incidence and types of cracks in bone. Lee et al. demonstrated a loss of compressive strength of porcine trabecular bone from freezing. However, while there was a significant difference in compressive strength after 5 years of freezing, there was no significant difference if the bone was only frozen for 1 year (47). All bones in our study were frozen for <1 year and our paired design allowed a relative comparison, regardless of any confounding factors.

We conclude that since the ratio of load to failure of novel TPLO constructs paired with predicate device constructs was significantly greater under the unique loading conditions of this experiment, mechanical adequacy is demonstrated and further evaluation is warranted. Future studies could include mechanical testing under cyclic loading conditions as well as study of the biological effects (healing time, SSI rates, etc.) of the novel titanium alloy TPLO plate/monocortical screw construct.

## Data Availability Statement

The original contributions generated for the study are included in the article/supplementary material, further inquiries can be directed to the corresponding author/s.

## Ethics Statement

Ethical review and approval was not required for the animal study because all specimens were cadaveric limbs from animals euthanized for reasons unrelated to the study.

## Author Contributions

SB was involved in testing and manuscript preparation. RP and NM performed cadaveric TPLO procedures and assisted in manuscript preparation. KM assisted in experimental set up and testing and assisted in data handling and manuscript preparation. ST was involved in experimental disease, testing, and manuscript preparation. All authors contributed to the article and approved the submitted version.

## Funding

This study for mechanical testing was provided by Kyon AG, Zurich, Switzerland.

## Conflict of Interest

ST reports personal fees from Kyon AG, Zurich, Switzerland, outside the submitted work and involved in the experimental design of the study. In addition, he has a patent USP 8968368 and others issued. The remaining authors declare that the research was conducted in the absence of any commercial or financial relationships that could be construed as a potential conflict of interest.

## Publisher's Note

All claims expressed in this article are solely those of the authors and do not necessarily represent those of their affiliated organizations, or those of the publisher, the editors and the reviewers. Any product that may be evaluated in this article, or claim that may be made by its manufacturer, is not guaranteed or endorsed by the publisher.
